# Antibiotic resistance and adhesion properties of oral *Enterococci *associated to dental caries

**DOI:** 10.1186/1471-2180-11-155

**Published:** 2011-06-29

**Authors:** Bochra Kouidhi, Tarek Zmantar, Kacem Mahdouani, Hajer Hentati, Amina Bakhrouf

**Affiliations:** 1 Laboratoire d'Analyses, Traitement et Valorisation des Polluants de l'Environnement et des Produits, Faculté de Pharmacie, rue Avicenne 5000, Université de Monastir (Tunisie; 2 Laboratoire de Biologie moléculaire, Hôpital Régionale de Kairouan, (Tunisie; 3 Service de Médecine et chirurgie buccales Clinique hospitalo-universitaire d'Odontologie, Université de Monastir (Tunisie

## Abstract

**Background:**

*Enterococci *are increasingly associated with opportunistic infections in Humans but the role of the oral cavity as a reservoir for this species is unclear. This study aimed to explore the carriage rate of Enterococci in the oral cavity of Tunisian children and their antimicrobial susceptibility to a broad range of antibiotics together with their adherence ability to abiotic and biotic surfaces.

**Results:**

In this study, 17 *E. faecalis *(27.5%) and 4 *E. faecium *(6.5%) were detected. The identified strains showed resistance to commonly used antibiotics. Among the 17 isolated *E. faecalis*, 12 strains (71%) were slime producers and 5 strains were non-producers. Among the 4 *E. faecium*, 2 strains were slime producers. All the tested strains were able to adhere to at least one of the two tested cell lines. Our result showed that 11 *E. faecalis *and 2 *E. faecium *strains adhered strongly to Hep-2 as well as to A549 cells.

**Conclusions:**

Drugs resistance and strong biofilm production abilities together with a high phenotypic adhesion to host cells are important equipment in *E. faecalis *and *E. faecium *which lead to their oral cavity colonization and focal infections.

## Background

*Enterococci *are normal commensals Gram-positive cocci that inhabit the gastrointestinal tract and the human oral cavity [1]. The increasing interest to *Enterococci *in clinical microbiology is linked to their high level intrinsic resistance to currently available antibiotics [2]. *Enterococcus faecalis *is responsible for up to 90% of human enterococcal infections [3]. However, *Enterococcus faecium *accounts for the remainder of infections caused by *Enterococci *spp. [1]. Data on oral prevalence of *E. faecalis *vary widely in different studies [4] which ranged from 0 to 50% depending on the oral source of the tested specimens (saliva, root canals, plaque) and the studied populations [5]. Sedgley et al., [4] reported the presence of *E. faecalis *in 29% of oral rinse samples and 22% in gingival sulcus samples collected from 41 endodontic subjects. Recently, drugs resistance in *E. faecalis *and *E. faecium *and their possible contribution to horizontal gene transfer underline the growing attention being paid to *Enterococci *in the oral cavity [6].

To date, *E. faecalis*, are not considered to be part of the normal oral microbiota [7]. However it has been considered as the most common species recovered from teeth with failed endodontic treatment [8] and to be the predominant infectious agent associated with secondary endodontic infections [9]. *E. faecalis *was shown to reside within different layers of the oral biofilm leading to failure of endodontic therapy [10]. These biofilms may contain up to several hundred bacterial species [11]. *Enterococci *in biofilms are more highly resistant to antibiotics than planktonically growing strains [12]. The possible role of adhesion and cells invasion as virulence factor associated with enterococcal infections has been reported [13]. Their capacity to bind to various medical devices has been associated with their ability to produce biofilms [14].

The attachment of different *E. faecalis *strains to several extracellular matrix proteins has been reported [15]. Bacterial adherence to host cells such as human urinary tract epithelial cells [16] and Girardi heart cells [17] was recognized as the initial event in the pathogenesis of many infections.

In view of the limited data, this study aimed to describe the Enterococci prevalence in the oral cavity of Tunisian children (caries active and caries free), their antimicrobial susceptibility to a broad range of antibiotics together with their adherence ability to abiotic and biotic surfaces.

## Methods

### Patients and Bacterial strains

The study was done on 62 children (34 caries active and 28 caries free) from the Dentistry Clinic of Monastir, Tunisia. The age group selected for the present investigation was about 4 to 12 years. Ethical clearance was taken prior to the commencement of study. Written informed consent was obtained from the parents of all participants. All clinical procedures were approved by the Ethical Committee of the Faculty of Medicine, Monastir University, Tunisia. A detailed medical and dental history was obtained from each parent. The criteria for inclusion were: no antibiotic treatment during the 4 weeks previous to sampling, no use of mouth rinses or any other preventive measure that might involve exposure to antimicrobial agents and no systemic disease.

Samples were taken from the oral cavity of each patient with a sterile swab. After incubation in brain heart infusion (BHI) medium during 2 h, the swab was plated on Bile Esculin Agar plates. Suspected colonies of *Enterococci *were tested for their positive Gram stain and catalase reaction (Oxoid, Basingstoke, UK).

Species identification was confirmed using API 20 Strep strips (Bio-Merieux, France) according to the manufacturer's recommendation and the results were read using an automated microbiological mini-API (Bio-Merieux, France).

### Molecular detection of oral *Enterococci*

Genomic DNA was extracted using a Wizard Genomic Purification Kit (Promega, Lyon, France). The presence of oral *Enterococci *was detected by polymerase chain reaction (PCR) using specific primers targeted for *E. faecalis*; E1, 5'-ATC AAG TAC AGT TAG TCT-3' and E2, 5'-ACG ATT CAA AGC TAA CTG-3'[18]. Primers for *E. faecium *EM1A, 5'-TTG AGG CAG ACCAGA TTG ACG-3' and EM1B, 5'-TAT GAC AGC GACTCC GAT TCC-3' [19]. PCR mixture (25 μl) contained 1 mM forward and reverse primers, dNTP mix (10 mM each of dATP, dCTP, dGTP and dTTP), 1 U of GO *Taq *DNA polymerase (Promega, USA), 5 μl green Go *Taq *buffer (5X), and DNA template (50 ng).

PCR products (5 μl) were analyzed on 1% (wt/v) agarose gel stained with ethidium bromide (0.5 μg/μl), visualized under ultraviolet transillumination and photographed using gel documentation systems InGenius (Syngene, USA).

### Antimicrobial susceptibility testing

Susceptibility to antibiotics was determined using the disc diffusion assay on Muller Hinton agar plates supplemented with 5% defibrinated sheep blood, according to the "Comité de l'antibiogramme de la Société française de microbiologie" [20]. using the following antibiotics (diffusible amount): PenicillinG (10 UI), Amoxicillin (25 μg), Ampicillin (10 μg), Amoxicillin/Clavulanic acid (20/10 μg), TIC: Ticarcillin (75 μg), Cefalotin (30 μg), Cefsulodin (30 μg), Ceftazidime (30 μg), Amikacin (30 μg), Gentamicin (500 μg), Kanamycin (1000 μg), Tobramycin (10 μg), Streptomycin (500 μg), Erythromycin (15 UI), Lincomycin (10 μg), Bacitracin (10 UI), Colistin (10 μg), Trimethoprim-Sulfamethoxazole (1.25/23.75 μg), Nalidixic acid (30 μg), Ciprofloxacin (5 μg), Ofloxacin (5 μg), Nitroxolin (20 μg) and Vancomycin (30 μg).

After 18 h of incubation at 37°C, inhibition zone diameters around each disc were measured and the strains were categorized as resistant, intermediate resistant, or susceptible to the antimicrobial agents based on the inhibition zone size [20].

### Phenotypic characterization of bacteria-producing slime

Qualitative Biofilm formation was studied by culturing strains on Congo red agar plate (CRA) made by mixing 36 g saccharose (Sigma Chemical Company, St. Louis, MO) with 0.8 g Congo red in one litre of Brain heart infusion agar (Biorad, USA) and incubated at 37°C for 24 h under aerobic conditions [21].

Results were interpreted as follows: Very black, black and almost black colonies on CRA, were considered to be normal slime-producing strains, while very red, red and bordeaux were classified as non-slime-producing strains [22].

### Semi quantitative adherence assay

Quantitative Biofilm production by the isolated strains was determined using a semi-quantitative adherence assay as described previously [13,23].

An overnight culture grown in BHI at 37°C was diluted to 1:100 in BHI with 2% glucose (w/v). A total of 200 μl of these cell suspensions was transferred in a U-bottomed 96-well microtiter plate (Nunc, Roskilde, Denmark). Wells with sterile BHI alone was served as negative control. Each strain was tested in triplicate.

The plates were incubated aerobically at 37°C for 24 h than the microtiter wells were washed twice with phosphate-buffered saline (PBS) and dried. Adherent bacteria were fixed with 95% ethanol and stained with 1% (w/v) crystal violet solution (Merck, France) for 5 min. The microplates were washed, air-dried and the optical density of each well was measured at 570 nm (OD_570_) using an automated Multiskan reader (GIO. DE VITA E C, Rome, Italy).

Biofilm formation was interpreted as follows: -: non-producer (OD_570_ < 0.120); +: weak producer (0.120 < OD_570_ < 0.240; ++: producer (0.240 < OD_570_ < 0.5) and +++: high producer (OD_570_ > 0.5) [24].

### Adherence to human epithelial cells

Human epidermoid carcinoma epithelial cells (Hep-2; ATCC CCL-23) and the respiratory epithelial cell line (A549) were cultured in Dulbecco's modified Eagle medium (DMEM) supplemented with 10% foetal calf serum (GIBCO-BRL) containing 1% penicillin (5 μg/ml) and streptomycin (100 μg/ml) and incubated with 5% CO_2 _at 37°C.

Cells (Hep-2 and A549) were seeded at a density of 5 × 10^5 ^/ml on glass coverslips placed in 24-well plates. All experiments were performed at 85-90% confluent cell monolayers. Prior to each experiment, the monolayer was washed with PBS and incubated with DMEM medium without antibiotics for 24 h. Overnight bacterial cultures were diluted at 1/100 into BHI broth and incubated at 37°C with agitation for approximately 2 h until the bacteria reached mid-log-phase. An aliquot of 100 μl of bacterial suspension of a density corresponding to approximately 2 × 10^6 ^CFU/ml was added to each cell. After incubation at 37°C for 3h, the coverslips were washed three times with PBS, fixed with methanol for 20 min, stained with Giemsa solution for 20 min and washed three times with PBS. Bacterial adherence to the cells was determined by light microscopy.

For each coverslip, a minimum of 800 cells was inspected to determine the percentage of infected cells, and next, 60-100 cells with bacteria were inspected to assess the number of cell associated bacteria. For each strain, two independent experiments were performed with two coverslips each [25]. Uninfected cells were included as a negative control.

### Statistical analysis

Statistical analysis was performed on SPSS v.17.0 statistics software. Pearson's chi-square χ2 test was used to assess inter-group significance. In addition Statistical significance was set at *P *< 0.05.

## Results

### Molecular identification of oral *Enterococci*

**Figure 1:**
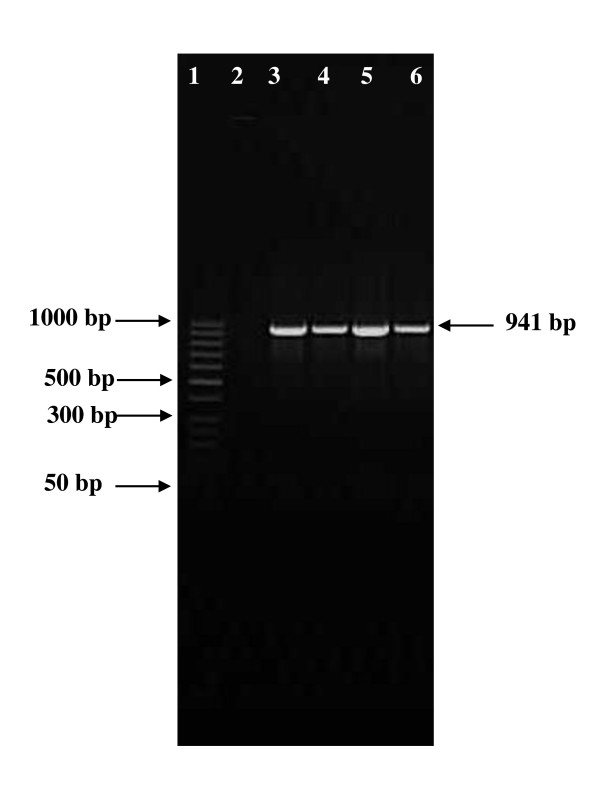
**Agarose gel electrophoresis of polymerase chain reaction (PCR) amplification of *Enterococcus faecalis *gene**. Lane 1 and 6: 25 bp DNA molecular size marker; Lane 2, negative control; lanes 3 to 6, PCR amplicons obtained with DNA amplification of *Enterococcus faecalis*: lane 3, B54; lane 4, B9; lane 5, B310; lane 6, B403.

**Figure 2:**
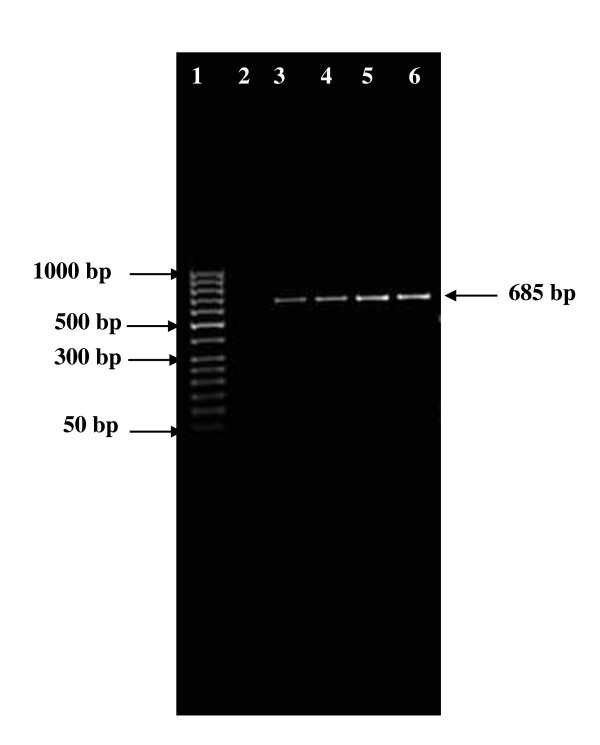
**Agarose gel electrophoresis of polymerase chain reaction (PCR) amplification of *Enterococcus faecium *gene**. Lane 1 and 6: 50 bp DNA molecular size marker; Lane 2, negative control; lanes 3 to 6, PCR amplicons obtained with DNA amplification of *Enterococcus faecium*: lane 3, B333; lane 4, B346; lane 5, B577; lane 6, B215.

In this study 113 Gram positive cocci were isolated from the oral cavity of 62 Tunisian children. Molecular identification using specific primer showed the presence of 17 *E. faecalis *giving a 941 DNA base pair product upon amplification (Figure 1) and 4 *E. faecium *giving a 658 DNA base pair product (Figure 2).

Consequently, the prevalence of *E. faecalis *and *E. faecium *were 27.5% (17/62) and 6.5% (4/62) respectively (Table 1).

**Table 1:** Antimicrobial susceptibility of the oral *Enterococci*

Antibiotics		No. (%)^a ^of resistant strains		
	
		*E. faecalis *(n = 17)	*E. faecium *(n = 4)	Total (n = 21)
PENICILLINS	P	17 (100)	4 (100)	21 (100)
	Amx	6 (35)	0(0)	6 (29)
	AM	6 (35)	1 (25)	7 (33)
	AMC	4 (25)	1 (25)	5 (24)
	TIC	17 (100)	4 (100)	21 (100)
CEPHALOSPORINS	CF	0(0)	0 (0)	0 (0)
	CFS	17 (100)	4 (100)	21 (100)
	CAZ	17 (100)	4 (100)	21 (100)
AMINOGLYCOSIDS	AN	17 (100)	4 (100)	21 (100)
	GM	4 (25)	1 (25)	5 (24)
	K	5 (29)	0 (0)	5 (24)
	TM	17 (100)	4 (100)	21 (100)
	S	17 (100)	4 (100)	21 (100)
MACROLIDS	E	17 (100)	4 (100)	21 (100)
LINCOSAMIDS	L	17 (100)	4 (100)	21 (100)
POLYPEPTIDS	B	17 (100)	4 (100)	21 (100)
	CS	16 (94)	4 (100)	20 (95)
SULFAMIDS-TRIMETHOPRIME	SXT	12 (71)	3 (75)	15 (71)
GLYCOPEPTIDS	VA	0 (0)	0 (0)	0 (0)
QUINOLONES	NA	17 (100)	4 (100)	21 (100)
FLUOROQUINOLONES	CIP	17 (100)	4 (100)	21 (100)
	OFX	17 (100)	4 (100)	21 (100)
DIVERS	NI	17 (100)	4 (100)	21 (100)

P:PenicillinG, Amx: Amoxicillin, AM: Ampicillin, AMC: Amoxicillin/Clavulanic acid, TIC: Ticarcillin, CF: Cefalotin, CFS:Cefsulodin, CAZ: Ceftazidime, AN: Amikacin, GM: Gentamicin, K: Kanamycin, TM: Tobramycin, S: streptomycin, E: erythromycin, L: Lincomycin, B: Bacitracin, CS: Colistin, SXT: Trimethoprim-Sulfamethoxazole, VA: Vancomycin, NA: Nalidixic acid, CIP: Ciprofloxacin, OFX: Ofloxacin, NI: Nitroxolin.

In the carious group population, the prevalence of *E. faecalis *and *E. faecium *were 46.9% (15/32) and 9.5% (3/32). However, in the caries-free one, the prevalence of *E. faecalis *and *E. faecium *were 7% (2/28) and 3.5% (1/28) respectively.

### Antimicrobial susceptibility testing

The antibiotic susceptibility of the isolated oral *Enterococci *showed the presence of multiresistant strains (Table 1).

Resistance profiles of *Enterococci *to the antimicrobial agents were as follows: penicillin, ticarcillin, Cefsulodin, Ceftazidime, Amikacin, Tobramycin and Streptomycin, 100%; Colistin, 91%, Trimethoprim-Sulfamethoxazole, 71%, Ampicillin, 33%, Amoxicillin, 29%, Amoxicillin/Clavulanic acid, Gentamicin and Kanamycin, 24%. Furthermore all the strains were susceptible to Cefalotin and Vancomycin.

### Phenotypic characterization of bacteria-producing slime

Among the 17 isolated *E. faecalis*, 12 strains (71%) were slime producers developing almost black, black or very black colonies on the CRA plate and the remaining 5 strains were non-producers developing red or bordeaux colonies (Table 2).

**Table 2:** Biofilm formation and of oral *Enterococci *and their adherence to abiotic and biotic surfaces

Strains	Identification	Origin	Phenotypes on CRA	Slime production	Mean OD595 ± SD		*OD595	Adherence	
							
								Hep-2	A 549
B347	*E. faecalis*	Caries active	AB	Producer	0.152	0.003	+	Moderately	Moderately
B342	*E. faecalis*	Caries active	Black	Producer	0.955	0.045	+++	Strongly	Strongly
B358	*E. faecalis*	Caries active	Brd	Non-producer	0.224	0.008	+	Strongly	Strongly
B403	*E. faecalis*	Caries active	AB	Producer	0.360	0.011	++	Strongly	Strongly
B310	*E. faecalis*	Caries active	AB	Producer	0.853	0.009	+++	Strongly	Strongly
B281	*E. faecalis*	Caries active	AB	Producer	0.508	0.018	+++	Strongly	Strongly
B312	*E. faecalis*	Caries active	Black	Producer	0.750	0.008	+++	Strongly	Strongly
B345	*E. faecalis*	Caries active	AB	Producer	0.550	0.026	+++	Strongly	Strongly
B54	*E. faecalis*	Caries active	Black	Producer	0.367	0.052	++	Strongly	Strongly
B'381	*E. faecalis*	Caries active	Brd	Non-producer	0.429	0.002	++	Strongly	Strongly
B9	*E. faecalis*	Caries active	Brd	Non-producer	0.391	0.002	++	Strongly	Strongly
B366	*E. faecalis*	Caries active	Black	Producer	0.211	0.004	+	Moderately	Weakly
B362	*E. faecalis*	Caries active	Brd	Non-producer	0.261	0.017	+	Strongly	Moderately
B385	*E. faecalis*	Caries active	AB	Producer	0.244	0.075	+	Strongly	Moderately
B361	*E. faecalis*	Caries active	AB	Producer	0.290	0.249	+	Moderately	Moderately
B368	*E. faecalis*	Caries free	Brd	Non-producer	0.202	0.008	+	Strongly	Strongly
B412	*E. faecalis*	Caries free	AB	Producer	0.291	0.011	+	Strongly	Moderately
B336	*E. faecium*	Caries active	Red	Non-producer	0.228	0.001	+	Strongly	Strongly
B346	*E. faecium*	Caries active	Brd	Non-producer	0.181	0.003	+	Moderately	Moderately
B577	*E. faecium*	Caries active	Very Black	Producer	0.179	0.035	+	Moderately	Moderately
B215	*E. faecium*	Caries free	AB	Producer	1.238	0.011	+++	Strongly	Strongly

*Biofilm production: -: non-producer (OD_570 _< 0.120); +: weak producer (0.120 < OD_570 _< 0.240; ++: producer (0.240 < OD_570 _< 0.5); +++: high producer (OD_570 _> 0.5).

Among the 4 *E. faecium*, 2 strains were slime producers developing almost black (B215) and very black colonies (B577) on the CRA plate.

### Semi quantitative adherence assay

All the examined strains were biofilm producers using the semi quantitative adherence assay (Table 2) and the OD_570 _were above 0.12, i.e. the value recognized as the limit under which strains were considered non-producers [24]. Six isolates showed an OD_570 _higher than 0.5 (indicated as +++ in Table 2), this being the threshold for strongly biofilm producers.

### Adherence of oral *Enterococci *to Hep-2 and A549 cells

Here, we analyzed the ability of *Enterococcus *strains isolated from oral cavity to adhere to the human epidermoid cancer (Hep-2) and the human lung adenocarcinoma epithelial (A549) cell lines. All the tested strains are able to adhere to at least one of the two tested cell lines.

Our result showed that 11 *E. faecalis *and 2 *E. faecium *strains adhered strongly to Hep-2 as well as to A549 cells (Table 2). Two strains were moderately adherent to both cells lines. In addition three strains were strongly adherent to Hep-2 cells while moderately adherent to A549 cells (Table 2).

## Discussion

In the last decade, several studies have focused on the relationship between periodontal diseases and oral bacteria. The current investigation examined the prevalence of *Enterococci *in the oral cavity of Tunisian children using specific primers.

In this study, 21 *Enterococci *(33.9%) among 113 Gram positive cocci were isolated and identified from the oral cavity of 62 children. Nineteen *Enterococci *were isolated from carious lesion (55.8%) and two from caries free (7%). Similar results have been reported by Gold et al., [5] suggesting that *Enterococci *were detected in 60% of oral samples collected from carious school children.

Data presented in table 1 showed a significantly higher frequency of *E. faecalis *(n = 17) than *E. faecium *(n = 4). This result was contradictory with a recent study reported a low prevalence rate of *E. faecalis *(3.5% to 13.5%) in intraoral sites [26].

Antimicrobial agents are frequently used in dentistry [27], which may however lead to drug resistance among the other oral bacteria [28]. In this study, the isolated strains were examined for their antimicrobial susceptibility to a broad range of antibiotics. Our results revealed the presence of resistant *Enterococci (E. faecalis *and *E. faecium*) to a wide range of antibiotics such as penicillin, Ticarcillin, Cefsulodin, Ceftazidime, Amikacin, Tobramycin, streptomycin, erythromycin, Lincomycin, Bacitracin, Nalidixic acid, Ciprofloxacin, Ofloxacin and Nitroxolin (Table 1). This is a serious problem, as it reduces the number of possible antimicrobial therapies for dental infections associated to *Enterococci*. Furthermore all the isolated strains were susceptible to Cefalotin and Vancomycin. Resistant *Enterococci *to currently available antibiotics pose real therapeutic difficulties [29] and can lead to the endodontic treatment failures result [30]. Moreover, transfer of resistance determinants from *Enterococci *to other more virulent Gram-positive bacteria, like staphylococci, has been observed in vitro [31]. Our previous data supported the presence of resistance oral streptococci [32] and the association of *Staphylococcus aureus *with dental caries [33] which carried various antibiotics and disinfectants resistance genes [34].

*E. faecalis *is responsible for endodontic infections due to their adherence to dentin collagen, and their resistance to endodontic therapy [26].

*Enterococci *are the third most common pathogen isolated from bloodstream infections and the most frequently isolated species in teeth with persistent infection after root canal treatment [35]. Different bacteriological studies have evaluated that *E. faecalis *is present in 29-46% of root-filled teeth with periapical lesions [36]. These findings highlight the ability of *E. faecalis *to persist in the post endodontic root canal environment [37]. One of the virulence factors that allow *Enterococci *to persist within the oral cavity is biofilm formation. Oral *Enterococci *produce virulence factors including aggregation substances, surface adhesins, lytic enzymes, and haemolysins [38]. The prevalence of biofilm positive *Enterococci *varied worldwide. Many studies have reported the ability of *Enterococcus *derived from various clinical origins to form biofilm [24]. Thus, biofilm formation may be an important factor in the pathogenesis of enterococcal infection.

Our data showed that 71% of *E. faecalis *and 50% of *E. faecium *were slimes producer on CRA plates. Moreover, all the examined strains were biofilm producers on microtiter plate (OD_570_ > 0.120). Statistical analysis revealed a correlation between the slime production on CRA and the semi quantitative adherence assay value (*P *< 0.001). Similar results have been reported by Arciola et al., [24] who confirmed that the majority of *E. faecalis *isolated from orthopedic implant-related infections are able to form biofilm.

Quantitative adherence determination showed a wide range of variation in adherence among strains, and the one sample-t test revealed a significant difference in adherence potency between the tested strains (*P *< 0.001).

A number of adhesion factors of *Enterococci *have been identified that confer binding to mucosal and other epithelial surfaces and facilitate host colonization [39]. Aggregation substance seems to mediate the specific binding of *Enterococci *to intestinal epithelium [40], renal epithelial cells [41], and macrophages [42] which increase their intracellular survival [42]. Since *Enterococci *are among the leading causes of endocarditis, and also exist as opportunistic bacteria in the oral cavity, bacterial adherence assay was performed to assess the binding efficiency of *Enterococci *to Hep2 and A549 cells.

All the isolated bacteria adhered to host cells. Among them16 and 13 strains were defined as strongly adherent to Hep-2 and A549 cells respectively (Table 2) confirming previous restudy suggesting the adherence ability of *Enterococci *to many host cells especially cardiac (GH), urinary tract epithelial cells (Vero, HEK) and intestinal cells [43].

At this point, we succeeded to establish a correlation between the semi quantitative adherence assay and the adherence potency to Hep2 and A549 cells (*P *< 0.001). The high adherence level of oral *Enterococci *to host cells increases their pathogenecity and confirms the role of the oral cavity as a reservoir of bacterial pathogens for focal infections.

## Conclusion

In summary, the oral cavity has been shown to be a reservoir for drug-resistant *Enterococci*. More importantly, our findings provide additional evidence for the persistence and adherence abilities of these bacteria within the carious lesions. The high rate of drugs resistance, strong biofilm formers and strong adherent to host cells *Enterococci *suggests that these three factors may play an important role in enterococcal infections. The establishment of such pathogen in the dental biofilm in addition to its multi-resistance, close attention should be given to these strains in order to reduce the risk for development of systemic diseases caused by *Enterococci *in other areas of the body.

## Competing interests

The authors declare that they have no competing interests.

## Authors' contributions

BK was the primary author of the manuscript, assisted in samples collection, molecular identification of oral Enterococci, antimicrobial susceptibility, biofilms and adherence assay.

TZ was the person contributed in biofilms assay and helped in the writing of the manuscript.

KM was the person who participated in data acquisition and contributed in writing the manuscript. HH helped in samples collection, designed and participated in the writing of the manuscript. AB provided funding, supervised the study, and helped to finalize the manuscript.

All authors read and approved the final version of the manuscript.

## Financial competing interests

Ministère Tunisien de l'Enseignement Supérieur, de la Recherche Scientifique'' through the ''Laboratoire d'Analyses, Traitement et Valorisation des Polluants de l'Environnement et des Produits, Faculté de Pharmacie, rue Avicenne 5000 Monastir (Tunisie).
